# Glyphosate: Its Environmental Persistence and Impact on Crop Health and Nutrition

**DOI:** 10.3390/plants8110499

**Published:** 2019-11-13

**Authors:** Ramdas Kanissery, Biwek Gairhe, Davie Kadyampakeni, Ozgur Batuman, Fernando Alferez

**Affiliations:** 1 University of Florida–IFAS, Southwest Florida Research & Education Center, 2685 State Road 29 N, Immokalee, FL 34142, USA; biwekgairhe@ufl.edu (B.G.); obatuman@ufl.edu (O.B.); alferez@ufl.edu (F.A.); 2 University of Florida–IFAS, Citrus Research & Education Center, 700 Experiment Station Rd., Lake Alfred, FL 33850, USA; dkadyampakeni@ufl.edu

**Keywords:** glyphosate, herbicide degradation, crop health, nutrient availability

## Abstract

Glyphosate-based herbicide products are the most widely used broad-spectrum herbicides in the world for postemergent weed control. There are ever-increasing concerns that glyphosate, if not used judiciously, may cause adverse nontarget impacts in agroecosystems. The purpose of this brief review is to present and discuss the state of knowledge with respect to its persistence in the environment, possible effects on crop health, and impacts on crop nutrition.

## 1. Introduction

Glyphosate (*N*-(phosphonomethyl) glycine), after its introduction in the 1970s, became a popular herbicide among farmers because of its broad-spectrum weed control. The use of glyphosate as a “burn down” application alone, or in combination with other pre- or postemergent herbicides, became standard practice in cropping systems throughout the world. Glyphosate is a nonselective, postemergent herbicide known to control more than 150 weed species, including mono- and dicotyledonous plants of annual or perennial nature [1]. Glyphosate is the active ingredient in many herbicide products (for example, Roundup) and is commercially available in its various salt forms, such as isopropylamine, ammonium, potassium, and trimesium salt. It is used to manage annual broadleaf weeds, grasses, and sedges in various field and row crops around the globe. Furthermore, its usage has expanded to urban and natural areas, pastures, forestry, and aquatics.

Generally applied to foliar parts of weeds, glyphosate can enter plants through four potential routes: the leaves or other green tissues, the roots, the trunk, or shoots emerging from the root or the trunk [2]. After entering the plants, it is rapidly translocated to regions of active growth within the plant. The mechanism of action of glyphosate is to block the activity of the enzyme called 5-enol-pyruvyl-shikimate-3-phosphate synthase (EPSPS), which catalyzes the sixth step in the shikimic acid pathway [3,4]. By blocking the enzyme, it prevents the biosynthesis of aromatic amino acids, viz. phenylalanine, tyrosine, and tryptophan, produced through the shikimate pathway [5]. Plants treated with glyphosate normally die within a period of 1–3 weeks, and because of its even distribution in the plant, no plant parts can survive [6].

Chemically, glyphosate is a phosphonomethyl derivative of the amino acid glycine [7]. It is a white and odorless crystalline solid having one basic amino group and three ionizable acidic sites (Table 1) [8]. Glyphosate is a nonvolatile chemical, does not undergo photochemical degradation, and is stable in air. Glyphosate has been considered a relatively safe compound in the environment because of its rapid inactivation in soil by adsorption and degradation [9]. However, owing to its extensive use, concerns and studies on the behavior of glyphosate in plant and the environment are growing.

**Figure 1:**
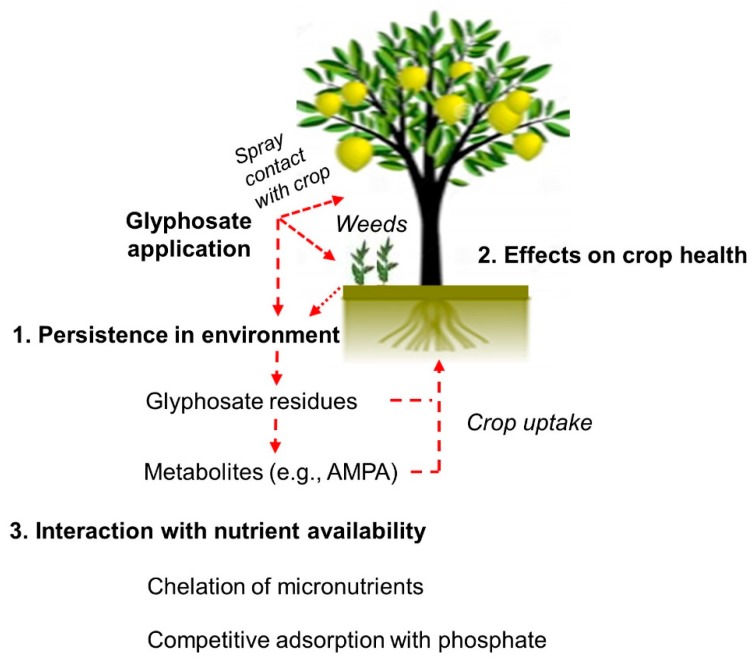
Schematic representation of the potential effects of glyphosate in crop production.

Especially due to improper application practices and excessive spray, the widespread presence of glyphosate has been observed in the aquatic and terrestrial environments [13]. In many studies, glyphosate has been detected in soil, crop products, animals that feed on crop products, humans, freshwater, and the organisms that live there [14]. Despite favorable evaluations of weed control efficacy and environmental risks of glyphosate, an increasing number of more recent observations suggest a relationship between extensive glyphosate application and adverse nontarget effects in agroecosystems [15]. The more significant among these concerns are (1) persistence in the environment, (2) effects on crop health, and (3) interaction with crop nutrition (Figure 1).

## 2. Glyphosate Persistence in the Environment

**Figure 2:**
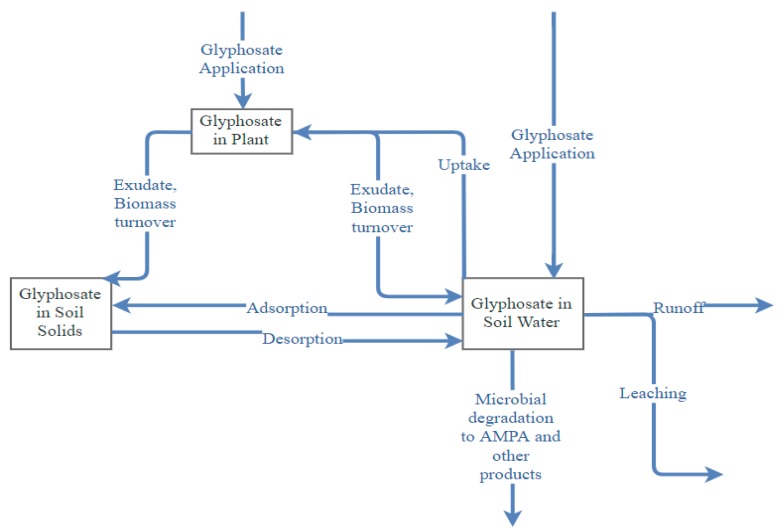
Fate and movement of glyphosate in different pools.

Applied as foliar spray to control weeds, glyphosate may end up in different soil pools and nontarget sites (Figure 2). Wash-off from the foliage or undirected spray drift [16], death and decay of glyphosate-treated plant residues, and exudation from the roots [17] may transport glyphosate to the soil. The release of glyphosate may even occur as exudates from undamaged roots of glyphosate-tolerant crops [18].

Glyphosate has an affinity to bind to soil particles and thus mostly accumulates in the top-soil layers. Processes like surface runoff, drift, and vertical transport in soil may transport it to groundwater, surface water, and water sediment [19,20,21]. The mobility and leaching of glyphosate have been tested in laboratory, lysimeter, and field conditions [11]. In a study on glyphosate leaching and movement conducted in a field site in Denmark, glyphosate, despite its high binding tendency on soil, was found to transport deep into the soil and leach out with drainage water [22]. Furthermore, there are several water monitoring reports that provide information on the occurrence of glyphosate in groundwater. Glyphosate was detected in 36% of a total of 154 water samples collected from Midwestern U.S. states, where glyphosate is extensively used on corn [23]. However, the glyphosate concentration in the detected samples was well below the maximum contaminant level for this herbicide. Beyond its presence in the groundwater, glyphosate has also been detected in surface water [24,25,26]. The predominant occurrence of glyphosate in surface water could be potentially attributed to surface water runoff [11]. Owing to extensive usage, this chemical may pose chronic and remote hazards to the ecological environment [27]. The major route of degradation of glyphosate from soil is microbial-mediated degradation or biodegradation [28].

Glyphosate degradation is a mainly microbial-mediated process [29,30], and the pathway has been widely studied in laboratories [31]. It degrades at a relatively rapid rate in most soils, with half-life estimated between 7 and 60 days [12]. Many studies have indicated that the presence of glyphosate in the soil can enhance microbial activity [32,33], while some studies have also shown the toxic effects of glyphosate on soil microorganisms [34].

The extent and rate of glyphosate biodegradation are influenced by processes such as adsorption and desorption in soil, along with other chemical, physical, and biological factors. Both aerobic and anaerobic conditions favor the degradation of glyphosate, even though anaerobic degradation is generally slower than aerobic degradation [35]. Similarly, soil temperature can also play an important role in determining glyphosate degradation [36]. The rate of mineralization of glyphosate was found to be correlated with the abundance of *Pseudomonas* spp. in soil by Gimsing et al. [30]. They also found that the addition of phosphate in the soil stimulates glyphosate mineralization. Lancaster et al. [37] compared the amount of ^14^CO_2_ production from mineralization of ^14^C-glyphosate in single herbicide application versus repeated applications. They found reduced production of ^14^CO_2_ from multiple applications, suggesting that long-term herbicide treatment did not favor acclimation of glyphosate-mineralizing microorganisms.

Glyphosate appears to be biodegraded cometabolically [38] as microorganisms are not able to utilize it as a source of carbon [39]. Cometabolic involvement of microbes in the degradation of this chemical is also denoted by the fact that glyphosate degradation and general microbial activity in the soil are correlated. Another evidence presented for cometabolic degradation of glyphosate is the absence of lag phase in soil [28], which implies that the degrading enzymes must already be present in the soil before glyphosate application. On the contrary, a few studies have shown that microbes can utilize glyphosate as a substrate for carbon [33,40], phosphate [39], or nitrogen [32].

Degradation or mineralization of glyphosate has been found to have a negative correlation with the soil adsorption capacity for glyphosate [41], possibly because of low bioavailability. Despite being highly water-soluble, glyphosate has limited movement within the soil profile because of strong adsorption to soil particles [42]. Adsorption of glyphosate to soil is determined by the amount of clay, organic matter, and iron and aluminum oxides present in soil [43,44]. Soil processes, such as adsorption/desorption, may control the glyphosate degradation rate as strong adsorption by soil solids, such as iron and aluminum oxides, may prevent microbial access to the compound [45,46]. There have been several studies on the adsorption characteristics of glyphosate, but only a few have studied the effect of adsorption on glyphosate bioavailability in soil. Sorensen et al. [41] found limited bioavailability of glyphosate in higher depths of sandy soil profile, where high adsorption and low desorption of glyphosate corresponded with negligible mineralization. On the other hand, in a study by Schnurer et al. [47], adsorbed glyphosate was found to be microbially degradable, even though the microbial activity was reduced in the presence of the herbicide.

Glyphosate degradation by microbial activity has been broadly studied, and bacterial species involved in the degradation have been isolated and characterized [48]. Bacteria are considered to be the main drivers behind its degradation in soil, even though the fungi have also been found to play an important role [49]. Degradation studies of glyphosate as a source of phosphorus (P) in the pure culture and soil media seem to show differences in the degradation kinetics. Furthermore, the rate of glyphosate degradation also varies when different microorganisms are used [50]. A slow lag phase followed by accelerating phase was observed in the degradation of glyphosate by a pure culture, while no lag phase was seen in the soil [50]. Results from such studies imply that pure culture studies may yield important information on degrading potential of microbes, but the application of such information to in situ conditions requires further investigations.

Primarily, there are two pathways of microbial degradation of glyphosate [39]. In one pathway, the intermediate compound formed is aminomethylphosphonic acid (AMPA), and in the other, sarcosine and glycine are formed. However, AMPA is considered to be the most common metabolite of glyphosate degradation as it accounts for more than 90% of the reported metabolites. The enzyme glyphosate oxidoreductase breaks the C–N bond in glyphosate to produce AMPA and glyoxylate [51]. The bacterial enzyme glyphosate oxidoreductase employs flavine adenine dinucleotide (FAD) as a cofactor, which is crucial in the degradation pathways of glyphosate. The FAD is believed to be reduced at the active site by glyphosate. Glyphosate oxidoreductase enzyme is inserted into the plant genomes for making glyphosate-tolerant Roundup Ready^®^ crops [52].

## 3. Glyphosate’s Effects on Crop Health

Among several concerns pertaining to unintended effects of glyphosate, its negative effects on nontarget plants are of serious concern among producers. Glyphosate applied to control weeds can reach the nontarget areas through several routes. The primary route is through undirected spray applications or “spray drift”, which can directly carry the herbicide chemical to crops. Research has demonstrated that off-target movement or drift of glyphosate during application can be up to 10% of the applied rate in crops like soybean and cotton [16,53]. Although herbicide exposure during application drift would be considered sublethal, response can be potentially severe for susceptible crops. For instance, drift from glyphosate has been found to cause distorted fruit (often termed as “cat-facing”) to develop in tomatoes at sublethal rates of exposure [54].

Another potential route for glyphosate accumulation and stabilization in soils is represented by the release of glyphosate from plant residues of glyphosate-treated weeds. As glyphosate is fairly stable and not immediately metabolized in many plant species, substantial amounts can be extensively translocated to regions of active growth and accumulate, particularly in young tissues [55]. After weeds eventually die, it ends up in the soil following the decay of plant parts. More intensive evaluations have revealed that glyphosate is translocated within plants, accumulated in roots, and eventually released into the rhizosphere [56,57,58]. From the soil, glyphosate may also be reabsorbed by the target or nontarget plants back through the roots after the initial application. There are a few studies that have investigated the effects of root-zone exposure of glyphosate on crops, including cotton [59], maize [60], and rapeseed [61]. These studies indicate there is a likelihood for glyphosate’s root absorption into crops. However, most of the conclusions were drawn from observations in hydroponic nutrient solutions, and hence additional research would be valuable for better understanding the uptake of glyphosate from soils and its ensuing effects on crop functioning.

Glyphosate blocks the synthesis of essential amino acids through binding and subsequent inactivation of an enzyme (EPSPS) that is critical in the shikimate pathway [28]. An array of phenolic compounds that play a significant role in plant immunity are derived from the same metabolic pathway. By disrupting the synthesis of such defense compounds in plants, glyphosate predisposes the crops to attack by soil-borne pathogens [62]. Hence, it could be argued that continuous crop exposure to glyphosate may increase plant susceptibility to diseases [15,63]. Excessive glyphosate application has been linked to disease development in many crops. For instance, glyphosate applications were found to be the main factor in the development of diseases such as Fusarium head blight in agronomic crops [64]. There are documented reports of increased colonization of pathogen in wheat and barley roots correlated with burndown applications of glyphosate before planting [65]. Moreover, the effects of sublethal doses of glyphosate on perennial plants sometimes take a year after exposure to appear and continue for two or more years [66]. Glyphosate can also predispose plants to diseases indirectly by reducing the overall growth and vigor of the plants, modifying soil microflora that affects the availability of nutrients required for disease resistance, and altering the physiological efficiency of plants.

The root uptake and translocation of glyphosate in nontarget plants have been studied. In one such experiment to understand the consequences of glyphosate residues on plant species used in ecological restoration, test plants were grown in nonadsorbing media continuously treated with glyphosate. Observations suggested that nonadsorbed glyphosate residues can cause potential phytotoxicity to sensitive plants through root uptake and subsequent translocation to other parts of the plant [67]. However, the study system utilized in this work is comparable to a spray application situation that has a risk of high herbicide delivery rate, regardless of the label recommendation. The uptake, translocation, and metabolism of glyphosate in nontarget tea plants were examined in a hydroponic system by Tong et al. [68]. The highest content of glyphosate was observed in the plant roots, where it was also metabolized to AMPA. The glyphosate and its metabolite were transported from the roots through the xylem or phloem to the stems and leaves. The results from this study indicated that plant-available glyphosate could be continuously absorbed by roots, metabolized, and transported into edible tea leaves [68]. Glyphosate uptake into nontarget plants is suggested when the herbicide and its degradation products (e.g., AMPA) are found in plant tissues and seeds of crops like soybean and corn [69] and tree foliage [20] following application of glyphosate to manage weeds in farms and adjacent areas.

Another potential side effect of glyphosate that needs to be discussed is its effect on root formation. Bott and coworkers [70] demonstrated glyphosate’s ability to inhibit root elongation, lateral root formation, and root biomass production in soybeans. It was even demonstrated that glyphosate released from dead weeds could be absorbed through the roots of growing citrus plants [17]. After entering the plant system, glyphosate is rapidly translocated to young growing tissues of roots, where it can accumulate and inhibit growth [71]. By blocking the production of tryptophan, glyphosate prevents the synthesis of a major growth promoter called indole acetic acid (IAA), which can explain the reduction in root growth of plants [15].

There are also some concerns about the deleterious effects of glyphosate on fruit retention in tree crops, such as citrus. Fruit drop in citrus is a natural phenomenon, but an increase in fruit drop has been reported after glyphosate application, especially in late summer and fall for early-season oranges and grapefruits [72,73] with an impact on fruit yield. The reason for this glyphosate-linked drop is far from understood as it is not even consistent across different seasons. However, it is known that glyphosate enhances ethylene production in plant tissues, and ethylene exposure of mature citrus fruit may result in early abscission and fruit drop. More research is needed to understand the causes of this fruit drop and the exact role of glyphosate in this process.

## 4. Glyphosate’s Interaction with Crop Nutrition

Glyphosate’s interaction with soil occurs when a foliar spray hits the soil surface or when glyphosate is released from decomposing weed tissue [17]. Glyphosate in the soil will be immobilized by adsorption or binding to the soil colloids and hence persists in the soil. The adsorption characteristics of glyphosate are different from most other herbicides. Adsorption of glyphosate on the soil is influenced more by soil minerals rather than organic matter [74]. Glyphosate is a divalent metal cation chelator and has been purported to reduce the uptake and translocation of nutrients in crops. Recent evaluations on the chelating ability of glyphosate highlighted it as a key factor in nutrient deficiencies in crops. These reduced availabilities of nutrients as a result of external (in the soil) or internal (in the plants) interaction of glyphosate with cationic nutrients are observed in production systems that heavily rely on glyphosate for weed management. For instance, Eker et al. [75] found that glyphosate residues or drift may reduce the uptake and translocation of micronutrients, such as Mn and Fe, in nontarget plants and suggested glyphosate−metal complex formation in plant tissues and/or plant rhizospheres. These poorly soluble chelated complexes of glyphosate with micronutrients hinder their root uptake and translocation by the crops. There are many similar studies that link the ability of glyphosate to inhibit the acquisition of micronutrients, such as Mn, Fe, Zn and B, in plants exposed to glyphosate, either through spray drift [76,77] or root uptake [78]. Such interactions of glyphosate with plant nutrition may potentially pose consequences on crop health. For instance, in tree crops like citrus, it is well known that these micronutrients are involved in disease, particularly Huanglongbing (HLB), resistance mechanisms [79,80].

The mechanism of binding of glyphosate and phosphate compounds to the soil solids and adsorption sites have been found to be similar [81]. Thus, the mobility of P in the soil is affected by the presence of glyphosate. The interaction between glyphosate and P in soil was reported shortly after the herbicide was launched into the market [20]. Many of the studies conducted later have verified that P and glyphosate compete for adsorption in the soil, and the competition substantially differs in various kinds of soils [75,82,83]. Therefore, the competition between glyphosate and P for adsorption sites in soil seems to be vital and makes a significant impact on mobility and crop availability aspects of P as a crop nutrient. Unfortunately, there is sparse information in the literature that demonstrates the noteworthy effect of such competition on P nutrition of crops, and thus further investigation is required.

## 5. Conclusions and Future Direction

Glyphosate has often been termed as a “once-in-a-century herbicide” because of its tremendous impact on weed management and the crop production industry. Although known to degrade relatively quickly in the soil following application, glyphosate and its metabolites can possibly persist in soil, water, and plant tissues in certain conditions. Research suggests that glyphosate may reach groundwater, surface water, and several other nontarget sites through processes such as leaching and surface runoff. It is also evident from several studies that glyphosate applied to cropping systems can potentially reach unintended areas and plant tissues through processes like off-target herbicide movement, spray drift, and root uptake. While such exposure of crops to glyphosate would be considered sublethal, it would seem wise to comprehend the consequent impacts on the health and nutrition of crops.

The best way to prevent these adverse crop effects related to glyphosate use is to avoid the “off-target” movement or “spray drift” of this herbicide to unintended areas from the application site. Furthermore, soil analysis for residual content of glyphosate is beneficial to detect whether the affected soils contain herbicide residues above the threshold that leads to root uptake and related crop effects. Clearly, further research is needed to understand crop risks related to glyphosate residues in soils, particularly in soil settings with low adsorption capacity and at very high rates of herbicide application.

Owing to the relatively high mobility of glyphosate, the likelihood of a rise in surface and groundwater content in tandem with herbicide use is high. Hence, potential routes of exposure into the environment, as well as the consequent implications on animals and humans, need to be explored more thoroughly. Moreover, there is an increasing concern toward the existence and concentration of glyphosate residues in a variety of crops produced for human and animal consumption. This necessitates an advanced dietary risk assessment of glyphosate resulting from its exposure.

In a nutshell, the extensive use of glyphosate and the environmental risks associated with it warrant awareness among its users about its judicious utilization and necessitate further intense investigations to mitigate, avoid, or remove the problems resulting from its use.

## Figures and Tables

**Table 1:** Selected physical and chemical properties of glyphosate.

Chemical structure	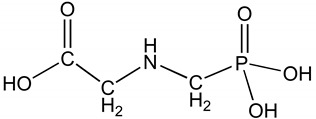
CAS number	1071-83-6
Chemical name	*N*-(phosphonomethyl) glycine
Empirical formula	C_3_H_8_NO_5_P
Molecular weight (g mol^−1^)	169.08
Water solubility (mg L^−1^ at 25 °C)	10,000 to 15,700 [10]
Octanol–water coeff. (*Kow*)	−4.6 to −1.6 [10]
Vapor pressure (mm Hg at 25 °C)	4.3 × 10^−10^ [10]
Freundlich adsorption coeff. (*Kads*) (L Kg^−1^)	0.6 to 303 [11]
Degradation half-life in soil (T1/2) (days)	7–60 [12]
Photolysis half-life (days)	Not substantial
EPA maximum contamination level (μg L^−1^)	700 [10]
